# Job satisfaction and associated factors among health professionals working at Western Amhara Region, Ethiopia

**DOI:** 10.1186/s12955-018-0898-7

**Published:** 2018-04-17

**Authors:** Kalkidan Temesgen, Moges Wubie Aycheh, Cheru Tesema Leshargie

**Affiliations:** 1 Emergency Department Case Team, Kuy Health Center, Kuy District, East Gojjam Zone, Amhara Region, Ethiopia; 2 Department of Public Health, College of Health Science, Debre Markos University, P.O. Box: 269, Debre Markos, Ethiopia

**Keywords:** Job satisfaction, Associated factors, Health professionals, Health institutions, Western Amhara, Ethiopia

## Abstract

**Background:**

In Ethiopia assuring the satisfaction of health care provider with their job is a major challenging problem. Job satisfaction is a worker’s emotional response to different job related factors resulting in finding pleasure, comfort, confidence, rewards, personal growth and various positive opportunities, including upward mobility, recognition, and appraisal done on a merit pattern with monetary value as compensation. Professionals, whose needs and expectations are satisfied, tend to be more productive compared to their colleagues. Thus, study is aimed at assessing job satisfaction and associated factors among health professionals working at Western Amhara region, Ethiopia.

**Methods:**

An institution-based cross sectional study was conducted on March 2016 at Western Amhara region among 575 health professionals selected using simple random sampling. Logistic regression analysis was used to identify factors related to job satisfaction. Variables which have *p*-value less than or equal to 0.05 with corresponding AOR at 95 confidence interval was considered to declare the significance association.

**Results:**

This study revealed that job satisfaction of health professional working at Western Amhara region was 31.7%. The mean age of respondent was 27.13 years. Majority of them, 79.3% and 95.3% were less than 30 years in age and orthodox Christian religion followers respectively. The presence of health professionals’ reference manual/guide, alcohol drinking, workload, experience, educational status and profession types were identified as significant factors associated with health care professionals’ job satisfaction level. Professional being laboratory technicians, pharmacists and Environmental health workers were 4.86 times more likely to satisfy themselves than nurses, midwives and Public health officers. Similarly, in their educational status, degree and above holders were 5.64 times more likely to satisfy themselves than below degree holders. Health professionals whose experience with > 3 years were 2.83 times more likely to satisfy themselves than counterpart. Health professionals who had high workloads were 3.99 times more likely to satisfy than those professionals whose workload was low. Professionals who did not drink alcohol were 3.55 times more likely to satisfy themselves than professionals who drank. Professionals who consult health reference manual/guide were 15.96 more likely to satisfy themselves than those professional who did not.

**Conclusion:**

Only one third of health professionals working at Eastern Amhara Region were satisfied on their job. The presence of health professionals’ reference manual/guide, alcohol drinking, workload, experience, educational status and profession types were identified as important predictors for job satisfaction.

## Background

Job satisfaction is an employee’s emotional response to different job-related factors resulting in finding pleasure, comfort, confidence, rewards, personal growth and various positive opportunities, including upward mobility, recognition and appraisal done on a merit pattern with monetary value as compensation [1, 2]. Variables that could satisfy employees differ from one to the other [3]. Any professional working in any organization has plenty of needs and expectations from the organization [4]. Professionals, whose needs and expectations are satisfied, tend to be more productive compared to their colleagues [5]. Health service delivery can be greatly affected by human resource [6]. Studies in Ethiopia showed that larger numbers of health professionals were not satisfied with their job. Low salary, limited educational development opportunity and inadequate facility and supplies were mostly described as a reason by study participants for their dissatisfactions [7].

Job satisfaction determined by a wide range of variables such as monthly salary, sufficient number of available staffs, comfortable working environment, training and growth opportunities, workload, supportive supervision provided, appreciation of good performers, timely evaluation, responsibility, relationship with the staffs and managers, job security, carrier development and other relevant behavioral and institutional factors [8].

Studies in Nepal [9], Serbia [10] and Pakistan [11] among health professionals showed that 24%, 77.6%, and 59% were not satisfied with their job respectively. Studies done at different parts of Africa showed that job satisfaction was low that means 47.9% in South Africa [2], 29%, in Malawi [2], 17.4% in Tanzania [2], 33.9% in Nigeria [1]. In addition, studies conducted in Ethiopia, job satisfaction among healthcare workers was only 58.6% in Jimma [12], 34.9% in West Showa zone [13], 53.8% in Dessie town [14].

In Vietnam among community health workers, age, areas of work and expertise, professional education, residence, sufficient number of staff were identified as factors affecting job satisfaction [15]. Hospital politics, personal relationships and the feeling of being able to provide a good quality of care also affects job satisfaction [10]. Moreover, age group, work experience, and position were also identified as factors affecting job satisfaction among healthcare workers in Vientiane capital and Bolikhamsai province, Lao PDR [16].

According to a study conducted in Jimma University (JU), sex, age, profession, service year, position and monthly salary were identified as factors affecting job satisfaction among health care providers [12]. A similar study conducted in West Shoa Zone, Oromia Regional State, Ethiopia indicated that scheme, lack of training opportunity, and lack of incentives, bureaucratic management style, poor performance evaluation system and poor working conditions were the factors which have an association with the poor satisfaction level of health professionals [13].

Besides, all the availed information regarding health workers job satisfaction on one part of the region there is in lack of it. Therefore, this study aimed at assessing the level of job satisfaction and associated factors among health professionals working in governmental health institutions in Western Amhara Region, Ethiopia.

## Methods

### Study design, study area, period and population

The institution-based cross-sectional study was used to conduct this study on March, 2016 at Western Amhara region, Ethiopia. The region is broadly divided as Eastern and Western region [17]. All health professionals working in Governmental Health Institutions of western Amhara region were considered as the study populations.

### Sample size determination

The sample size was determined by using a single population proportion formula considering the following assumptions: n = **(Z**_**σ/2**_**)**^**2**^**p (1-p)/ (d)** ^**2**^, Where: n = the required sample size, Z _α /2_ = 95% confidence interval (level of significance) (1.96), P = Expected proportion of the population (53.8%) [13], d = Desired precision /the margin of error (4%).

$$ n=\frac{(1.96)^20.538\left(1-0.538\right)}{(0.04)^2}n=597 $$

Ten percent was also added for non-response. Accordingly, the required sample size was computed and the final sample size was 657. Simple random sampling method was used to select 657 health professionals from the selected health institutions.

### Variable of the study

#### Dependent Variable

Job Satisfaction (Satisfied/Dissatisfied).

### Independent Variables

#### Socio-demographic characteristics

Age, Sex, religious status, marital status, educational status and family size,

#### Institutional characteristics

Workload, working hour, existence of health insurance and availability of manual for reference,

#### Behavioral characteristics

Khat chewing, cigarette smoking and alcohol drinking.

### Operational definition

#### Job satisfaction

for this research, health professionals were considered as satisfied with their job if they answered and got the score of 9 and above questions (greater than or equal to the mean value 50%) among 18 questions developed to assess the satisfaction level of respondents. We used the mean because the data was normally distributed.

#### Work load

In this research health professionals were considered as loaded if they gave service to more than 35 clients within a day [18].

### Data collection tool

A pre-tested and structured self - administered questionnaire was used to collect data. A questionnaire was adapted from different kinds of literature used in this study. Twenty Likert scale questions were used to measure job satisfaction.

### Data quality control

Both data collectors and supervisors were given for one-day training. The training was given in lecture, discussion, and role-play ways. Completeness of data was checked in every day of activity and the necessary feedback was offered to data collectors in the next morning. Besides this, the principal investigator and an experienced data clerk entered and cleaned the data before the commencement of the analysis.

### Data processing and analysis

The collected data was cleaned, coded and entered into Epi-data version 3.1 software and exported to SPSS version 20. Errors related to inconsistency were verified using cross tabulation and other data exploration methods. The results were presented in narrative, tables, and graphs. Furthermore, logistic regression, specifically bivariate and multivariate analysis was used to identify factors related to health professional job satisfaction. Those variables which were significant during bivariate analysis at *p*-value ≤ 0.2 were taken to multivariable logistic regression analysis. The adjusted odds ratios together with their corresponding 95% confidence intervals were computed. *P*-value ≤ 0.05 with 95% confidence interval was considered to declare a variable as a statistically significant with the dependent variable.

## Results

### Socio-demographic characteristics of respondents

A total of 575 participants were involved in the study with a response rate of 87.5%. The mean age of respondents was 27.13 years (SD = ±3.26 years). Slightly more than half 316 (55%) of the respondents were Male. The majority, 456(79.3%) and 548(95.3%) of them were in the age group less than 30 years and orthodox Christian in their religion respectively. A majority, 551(95.8%) were Amhara ethnicity and 339(59%) of the study participants were single in marital status. More than half (62.8%) of the study participants were diploma holders and 301(52.3%) of them were nurses (Table 1).

**Table 1:** Socio-demographic characteristics of job satisfaction of health professionals working in governmental Health institutions Western Amhara Region, Ethiopia, 2016 (*n* = 575)

Variable		Frequency	Percent
Sex	Male	316	55%
	Female	259	45%
Age	< 30 years	456	79.3%
	≥30 years	119	20.7%
Religion	Orthodox	548	95.3%
	Muslim	16	2.8%
	Protestant	11	1.9%
Ethnicity	Amhara	551	95.8%
	Oromo	18	3.1%
	Awi	6	1%
Marital status	Single	339	59%
	Married	236	41%
Profession	Physicians	40	7%
	Public Health officers	119	20.7%
	Nurses	301	52.3%
	Midwiferies	76	13.2%
	Others^a^	39	6.8%
Educational status	Diploma	361	62.8%
	Degree	214	37.2%
Service year	< 3 years	146	25.4%
	≥3 years	429	74.6%

^a^Laboratory, Environmental Health, Pharmacy professionals

### Behavioral characteristics of respondents

Among the total study participants, 32(5.6%) of them were Khat chewers and of whom 24(75%) and 8 (25%) of them were chewing three times per week and daily respectively. Eight (1.4%) of the respondents were cigarette smokers and all of them smokes half pack a day. Eighty (13.9%) of the study participants practiced alcohol drinking out of which 72(90%) and 8(10%) of them were drinking two/ three and more times respectively a week.

### Institutional characteristics of respondents

On average, three hundred eighty-five (67%) of the study participants provided service for less than 35 clients per working day. The majority, 414(72%) of the health professionals were working for less than or equal to 8 h per working day. Out of the total health care workers involved in this study, 544(94.6%) of them did not have health insurance. Four hundred twenty-three (73.6%) of the study participants did not have reference manual.

### Level of job Jatisfaction

The overall job satisfaction level of the health professional at governmental health facilities of western Amhara Region was 31.7% with 95% CI (27.7, 35.3). Among the study participants, 72(12.5%), 393(68.3%), 71(12.3%), 31(5.4%) and 8(1.4%) of them were strongly disagreed, disagreed, neutral, agreed and strongly agreed with the management style of their health institution respectively (Table 2).

**Table 2:** Level of agreement about factors affecting job satisfaction among health professionals working in governmental health institutions in Western Amhara Region, Ethiopia, 2016

Satisfaction related to Job	Degree of agreement				
	Strongly disagree	Disagree	Neutral	Agree	Strongly Agree
	*N* (%)	*N* (%)	*N* (%)	N (%)	*N* (%)
Staff relationship	40(7%)	252(43.8%)	31(5.4%)	236(41%)	16(2.8%)
Working environment	71 (12.3%)	376(65.4%)	40(7%)	80(13.9%)	8(1.4%)
Training opportunity	80 (13.9%)	433(75.3%)	16(2.8%)	46(8%)	0
Performance Evaluation	64 (11.1%)	447(77.7%)	16(2.8%)	48(8.3%)	0
Participate in decision making	48 (8.3%)	449(78.1%)	16(2.8%)	54(9.4%)	8(1.4%)
Clear job description	32 (5.6%)	447(77.7%)	16(2.8%)	64(11.1%)	16(2.8%)
Plan to leave the institution	64(11.1%)	383(66.6%)	24(4.2%)	96(16.7%)	8(1.4%)
Medical equipment supply	31 (5.4%)	408(71%)	48(8.3%)	80(13.9%)	8(1.4%)
Incentives or allowance	64 (11.1%)	455(79.1%)	24(4.2%)	32(5.6%)	0
Enabling condition	64 (11.1%)	463(80.5%)	16(2.8%)	32(5.6%)	0
Frequency of supervision	56 (9.7%)	456(79.3%)	32(5.6%)	31(5.4%)	0
Availability of transportation	56 (9.7%)	321(55.8%)	32(5.6%)	150(26.1%)	16(2.8%)
Government regulation	48 (8.3%)	447(77.7%)	16(2.8%)	48(8.3%)	16(2.8%)
Annual leave	64 (11.1%)	415(72.2%)	32(5.6%)	64 (11.1%)	0
Promotional strategies	64 (11.1%)	447(77.7%)	16(2.8%)	48(8.3%)	0
Carrier development	80 (13.9%)	415(72.2%)	16(2.8%)	64 (11.1%)	0
Annual increment of salary	104 (18.1%)	407(70.8%)	24(4.2%)	40(7%)	0
Location of the work	56(9.7%)	439(76.3%)	0	80 (13.9%)	0

### Factors associated with job satisfaction

In the bivariate analyses sex, age, profession, educational status, service year, workload, alcohol drinking, the existence of health insurance and the presence of reference manuals met the variable selection criteria (*P*-value < 0.2) and were fitted to multivariable logistics regression analysis. After fitting multivariate logistic regression model variables including types of a health professional, Educational status, service year, workload, drinking alcohol and Availability of the reference guideline were identified as significant factors for the satisfaction of health professionals on their job. Variables that were negatively associated with bivariate logistic analysis became significantly associated factors on the multivariate logistic regression when the confounding variable was controlled. Those health workers who categorized as others (environmental health, laboratory, and pharmacy) were 4.68 (AOR = 4.68, 95% CI (1.04, 20.9)) times more likely to get satisfied than health officers, nurses and midwives. Those health professionals with the degree and above holders were 5.64 times more likely to get satisfied with their job compared to those with an educational status diploma (AOR = 5.64, 95% CI (2.44, 13)). Health professionals who were serving in the institution for three and more years were 2.83 times more likely to get satisfied with their job compared to the counterpart (AOR = 2.83, 95% CI (1.39, 5.79)). Health professionals who were giving a service for 35 and more clients per day were 3.99 times more likely to get satisfied with their job compared to the counterpart(AOR = 3.99, 95% CI (2.13, 7.45)). Those health professionals who were not drinking alcohol were 3.55 times more likely to get satisfied with their job compared to those who were drinking alcohol (AOR = 3.55, 95% CI (1.31, 9.62)) and those health professionals who had access to reference manual were 15.96 times more likely to get satisfied with their job compared to those with no access to the reference manual (AOR = 15.96, 95% CI (8.49, 30)) (Table 3).

**Table 3:** Bivariate and multivariate analysis of factors affecting job satisfaction among health professionals working in governmental health institutions in Western Amhara Region, Ethiopia, 2016

Variables	Response	Job satisfaction		COR with 95% CI	AOR with 95%CI	*P*-value
		Satisfied	Dissatisfied			
		*N* (%)	*N* (%)			
Sex	Male	86 (27.1%)	230 (72.9%)	1	1	
	Female	96 (37.1%)	163 (62.9%)	1.57(1.10, 2.24)	1.09(0.62, 1.92)	0.747
Age	< 30 years	127 (27.5%)	329 (72.5%)	1	1	
	≥30 years	55 (46.2%)	64 (53.8%)	2.22(1.47, 3.37)	0.57(0.29, 1.14)	0.115
Profession	Physicians	32 (85.0%)	8 (15.0%)	1	1	
	PHO^*^	63 (52.9%)	56 (47.1%)	0.28(0.12, 0.66)	0.65(0.21, 1.96)	0.447
	Nurses	47 (15.6%)	254 (84.4%)	0.05(0.02–0.10)	0.27(0.07, 1.01)	0.052
	midwiferies	16 (21.1%)	60 (78.9%)	0.07(0.03, 0.17)	1.01(0.24, 4.24)	0.983
	Others**	24 (61.5%)	15 (38.5%)	0.4(0.15, 1.09)	4.68 (1.04, 20.9)	0.043*
Educational status	Diploma	71(19.7%)	290 (80.3%)	1	1	
	Degree	111(51.9%)	103 (48.1%)	4.4(3.03, 6.39)	5.64 (2.44, 13)	0.001*
Service year	< 3 years	24 (16.4%)	122 (83.6%)	1	1	
	≥3 years	158 (36.8%)	271 (63.2%)	2.96(1.83, 4.78)	2.83 (1.39, 5.79)	0.004*
Work load	Not loaded	79 (20.5%)	306 (79.5%)	1	1	
	Loaded	103 (54.2%)	87 (45.8%)	4.58(3.14, 6.69)	3.99 (2.13, 7.45)	0.001*
Drinking alcohol	Yes	16 (20.0%)	64 (80.0%)	1	1	
	No	166 (33.5%)	329 (66.5%)	2.01(1.13, 3.60)	3.55 (1.31, 9.62)	0.013*
Existence of health insurance	Yes	16 (51.6%)	15 (48.4%)	2.42(1.17, 5.02)	2.61(0.72, 9.40)	0.142
	No	166 (30.5%)	378 (69.5%)	1	1	
Availability of the reference guideline	Yes	104 (68.4%)	48 (31.6%)	9.58(6.29, 14.60)	15.96 (8.49, 30)	0.001*
	No	78 (18.4%)	345 (81.6%)	1	1	

^*^PHO=Public Health Officer, others^**^ = Environmental Health, Laboratory, Anesthetics, n = number, % = frequency

## Discussion

This institution-based cross-sectional study sought to determine the job satisfaction level and its associated factors at Eastern Amhara region of Ethiopia. The study found out that job satisfaction of health professional working in Western Amhara Region was 31.7%. This job satisfaction level is comparable with the two studies conducted in West Shewa Zone 34.9% [13] and Dire Dawa City administrative, Ethiopia (**34.5%)** [19].

In addition, this study revealed that the job satisfaction is lower than a study finding conducted in Ethiopia Jimma 58.6% [12], Dessie town 53.8% [14], federal police referral hospital, Addis Ababa, Ethiopia 43.2% [20]. Similarly, the current study result was lower compared with studies done outside of the country Ethiopia. These are 52.1% at South Africa, 71% in Malawi, 82.6% in Tanzania [2] 67.1% in Nigeria [1], 76% in Nepal, [9] and 41% in Pakistan [11]. This discrepancy might be because the previous studies were mainly urban-based while the current covers large geographic area in which majority was rural.

On the other hand, the current finding was higher than the finding revealed at 20.4% at South Rand Hospital of South Africa [21] and Serbia (22.4%) [10]. The discrepancies might be related to differences in the presence of reference manual and health insurance, monthly salary, education opportunity, and workload imbalance. Besides these, most health facilities selected in this study were remote and had poor infrastructure than the previous study. These might also cause the variation of the results.

Concerning about factors associated with job satisfaction, this study identified that the presence of guideline that helps professionals in referring, alcohol drinking status, workload, service year, educational status and types of professions are significantly associated with job satisfaction level of healthcare professionals.

Pharmacists, Environmental Health and Laboratory professionals were 4.68 times more likely to get satisfied with their job compared to Public health officers, Nurses, and Midwives. This finding is in line with the study conducted in Jimma [12].

Moreover, educationally health professionals with degree holder were 5.64 times more likely got satisfaction with their job compared with the diploma. This result is consistent with the finding from Vietnam [15]. Health professionals who were serving in the institution for ≥3 years were 2.83 times more likely got satisfaction with their job compared to< 3 years’ service. This result is in agreement with the finding from Vientiane capital and Bolikhamsai province, Lao PDR [16].

In addition to this, health professionals who were giving a service ≥35 clients per day were 3.99 times more likely to get satisfaction with their job compared to those with giving a service for < 35 clients per day. The possible reason for the finding might be related to the satisfaction they got from the service they provided for the patients who suffer from diseases.

Those health professionals who were not drinking alcohol were 3.55 times more likely to get satisfaction with their job compared to those who were drinking alcohol. The possible justification for the finding might be related to those who used to drink alcohol might get bored with their job not because of the job itself but rather because of the problem related to alcohol drinking.

Those health professionals who had access to the reference manual were 15.96 times more likely to get satisfied with their job compared to those with no access to reference manual. This might happen because professionals who had access to reference manual may perform their job as per the guideline and might get satisfaction with their job because clients who got the service as per the guideline may appreciate them and professionals might be motivated by the appreciation provided by the clients.

Concerning the gender of the respondents, even though it was not identified as a predictor variable in the multivariate analysis, female health professionals’ were more (37.1%) satisfied than their male colleagues (27.1%). This might be true that drinking alcohol was observed more frequently among male than female health professionals, and alcohol drinking was identified as significant factors with employee job satisfaction. A similar study conducted at Dire Dawa city administrative, Ethiopia suggested the similar satisfaction levels of male and female health professionals (33.8% for male and 33.6% for female, respectively) [19]. This finding was also similar to a study finding conducted at North Vietnam, which revealed that, females are more satisfied than the counterpart [22]. This might be because females have fewer problems with the behavioral characteristics. In contrary, a study conducted at Police Referral Hospital in Addis Ababa revealed 23.3% and the satisfaction levels observed between male (51.1%) and female (48.9%) [20] were the same.

## Conclusion

In conclusion, this study revealed that only one-third of the health professionals working in Eastern Amhara Region were satisfied on their job. The presence of health professionals’ guides to accomplish their activities, alcohol drinking, workload, experience, educational status and types of professions were identified as important predictors of job satisfaction. Furthermore, the presence of guideline that helps healthcare professionals, drinking alcohol, workload, service year, educational level and types of the profession were identified as factors that affect job satisfaction level of the study population. The concerned bodies should develop strategies on the identified factors to enhance the job satisfaction level of health care professional at western Amhara region, Ethiopia. Arranging opportunities for educational development is also one important strategy to enhance the workers satisfaction level.

## Abbreviation

AOR: Adjusted Odd Ratio
DMU: Debre Markos University
JS: Job satisfaction
JU: Jimma University
